# 636. Mortality Impact of Carbapenem-Resistant Acinetobacter baumannii (CRAB) Colonization and Infection: A Retrospective Cohort Study

**DOI:** 10.1093/ofid/ofaf695.200

**Published:** 2026-01-11

**Authors:** Regev Cohen, Shelly Lipman-Arens, Yael Galnoor-Tene, Linor Ishay, Olga Feld-Simon, Lamis Mahamid, Orna Ben-Natan, Aliza Vaknin, Mohammed Ganayem, Milena Pitashny, Alvira Zbiger, Rene Abilevitch, Said Younis, Elias Tannous

**Affiliations:** Technion University, Haifa, Netanya, HaMerkaz, Israel; Hillel Yaffe Medical Center, Hadera, HaZafon, Israel; Hillel Yaffe Medical Center, Hadera, HaZafon, Israel; Rappaport Faculty of Medicine, Technion, Haifa 3109601, Israel and Hillel Yaffe Medical Center, Hadera, Israel, Hadera, HaMerkaz, Israel; Hillel Yaffe Medical Center, Hadera, HaZafon, Israel; Hillel Yaffe Medical Center, Hadera, HaZafon, Israel; Hillel Yaffe Medical Center, Hadera, HaZafon, Israel; Hillel Yaffe Medical Center, Hadera, HaZafon, Israel; Hillel Yaffe Medical Center, Hadera, HaZafon, Israel; Hillel Yaffe Medical Center, Hadera, HaZafon, Israel; Hillel Yaffe Medical Center, Hadera, HaZafon, Israel; Hillel Yaffe Medical Center, Hadera, HaZafon, Israel; Hillel Yaffe Medical Center, Hadera, HaZafon, Israel; Hillel Yaffe Medical Center, Hadera, HaZafon, Israel

## Abstract

**Background:**

The clinical impact of carbapenem-resistant *Acinetobacter baumannii* (CRAB) remains controversial, with uncertainty about whether it directly contributes to mortality or merely reflects underlying patient severity. This study aimed to evaluate the impact of CRAB colonization and infection on patient outcomes while accounting for disease severity, acquisition timing and infection control interventions.

**Figure:**
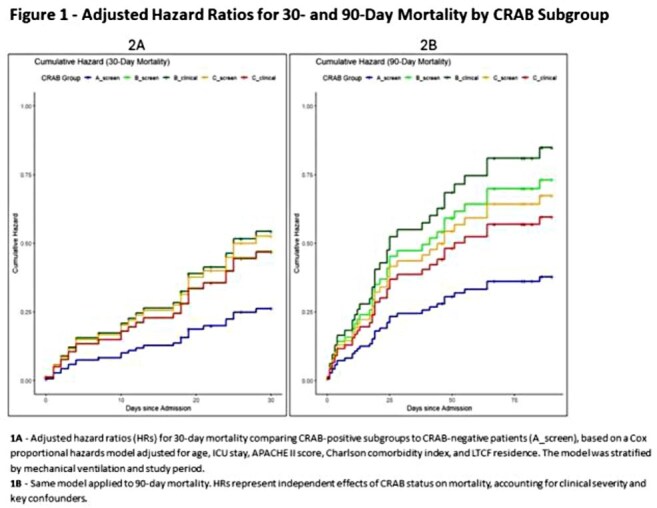
Adjusted Cumulative Hazard Ratios for 30- and 90-Day Mortality by CRAB Subgroup 1A - Adjusted hazard ratios (HRs) for 30-day mortality comparing CRAB-positive subgroups to CRAB-negative patients (A_screen), based on a Cox proportional hazards model adjusted for age, ICU stay, APACHE II score, Charlson comorbidity index, and LTCF residence. The model was stratified by mechanical ventilation and study period. 1B - Same model applied to 90-day mortality. HRs represent independent effects of CRAB status on mortality, accounting for clinical severity and key confounders.

**Methods:**

A retrospective cohort study of 3,080 patients in an Israeli hospital (January 2023-December 2024), categorized as CRAB-negative (A group), B group - CRAB-present on admission (POA) via screening only (B_screen) or clinical samples (B_clinical), and C group - hospital-acquired CRAB (C_screen and C_clinical). Infection control measures intensified during the study's late period – since July 2023. Adjustments were made for age, ICU stay, nursing home residency, ventilation, APACHE II and Charlson scores. Multivariable Firth logistic regression, propensity-score matching, and Cox proportional hazards analyses were used.

**Results:**

149 (4.8%) patients had CRAB POA and 108 acquired CRAB. Risk factors for CRAB-POA were nursing home and ventilation (OR=4.1 and 2.3, respectively). CRAB acquisition risk factors included increased length of hospital stay and ventilation. Both CRAB colonization and infection were associated with 30-day mortality after adjusting for confounders (B group: OR=3.22, C group: OR=2.17). These associations persisted at 90 days and in propensity-matched analyses. Cox models yielded hazard ratios of 1.79-2.07 for 30-day mortality across CRAB groups. Time-adjusted survival analyses demonstrated mortality increases shortly after CRAB acquisition. The late study period showed significantly reduced CRAB acquisition (OR=0.13) and mortality (OR=0.62) following implementation of enhanced screening and cohorting.

**Conclusion:**

CRAB colonization and infection is associated with nearly two-fold increases in 30- and 90-day mortality risk even after adjustment for disease severity scores, supporting its role as a direct contributor to adverse outcomes. Screening high-risk patients, and cohorting reduced CRAB acquisition and mortality.

**Disclosures:**

All Authors: No reported disclosures

